# Effect of inverted internal limiting membrane flap technique on small-medium size macular holes

**DOI:** 10.1038/s41598-021-04739-x

**Published:** 2022-01-14

**Authors:** Kanako Yamada, Akio Oishi, Mao Kusano, Hirofumi Kinoshita, Eiko Tsuiki, Takashi Kitaoka

**Affiliations:** Department of Ophthalmology and Visual Sciences, Graduate School of Biomedical Sciences, Nagasaki University, Sakamoto 1-7-1, Nagasaki, 852-8102 Japan

**Keywords:** Diseases, Medical research

## Abstract

Inverted internal limiting membrane (ILM) flap technique was developed to achieve macular hole (MH) closure in large MH and refractory cases. In this study, we evaluate the effect of the technique for small-medium size MH. We recruited patients who underwent vitrectomy for small-medium size (< 400 μm) MH with either inverted ILM flap technique (flap group) or with conventional ILM peeling (peeling group). Using propensity score, 21 eyes of 21 patients in the peeling group were matched against 21 eyes of 21 patients in the flap group. We compared MH closure rate, postoperative visual acuity, and recovery of the external limiting membrane (ELM) and ellipsoid zone (EZ). The MH closure rate was not different between the two groups (flap vs peeling: 90% vs 100%, P = 0.49). Whereas there was no significant difference in visual acuity improvement between the two groups, the flap group showed more disruption of the ELM 3 months after surgery and of the EZ at 3 and 6 months after surgery (P = 0.02, P = 0.03, and P = 0.04, respectively). The result suggested that inverted ILM flap technique does not have additional benefits for small-medium size MHs and may delay recovery of retinal integrity.

## Introduction

A macular hole is characterized by a full thickness central foveal hole and causes metamorphopsia and vision loss. Since Kelly et al.^1^ reported pars-plana vitrectomy (PPV) for treating macular holes (MHs) in 1991, it has become a standard treatment for MHs^2–6^. While the MH closure rate was improved with internal limiting membrane (ILM) peeling^7^, it was still suboptimal in large MHs^8^. In 2010, Michalewska et al.^9^ reported the inverted ILM flap technique for large idiopathic MHs. The technique consists of partial peeling of the ILM and placing the flap over the MH. The authors reported that the technique increased the rate of complete MH closure to 98% for large idiopathic MHs (> 400 μm) compared with 88% with conventional PPV and ILM peeling.

Although the inverted ILM flap technique was originally developed for large MHs, myopic MHs, and MH retinal detachment^10–13^, the technique is sometimes used for small-medium size MHs^14, 15^. The risks and benefits of this approach have yet to be determined.

In this study, the MH closure rate, postoperative best-corrected visual acuity (BCVA), and recovery of the external limiting membrane (ELM) and ellipsoid zone (EZ), which are closely associated with postoperative BCVA^16–20^, were compared in the inverted ILM flap technique and conventional ILM peeling in small-medium size MHs.

## Material and methods

This was a retrospective, nonrandomized, comparative study. The study was approved by the Nagasaki University Hospital Clinical Research Ethics Committee and complied with the Declaration of Helsinki. Information about this study was made public, and patients were given the opportunity to refuse to participate in this study. The ethics committee waived the need for written informed consent.

Patients who underwent vitrectomy for MH at Nagasaki University Hospital between July 2014 and October 2017 were recruited. Among the participants, patients whose minimum MH diameter was < 400 μm, full-thickness MH were included. Exclusion criteria were recurrent and secondary MHs, high myopia (axial length ≥ 27 mm), and MH with retinal detachment.

Standard 3-port 25-gauge pars plana vitrectomy was performed with Constellation Vision System (Alcon Surgical, Ft. Worth, TX, USA). Triamcinolone acetonide was used to visualize the vitreous. Selection of inverted ILM flap technique or conventional ILM peeling was according to the surgeons’ discretion. The ILM peeling (peeling group) consisted of peeling and complete removal of the ILM in a circular fashion around the MH. The inverted ILM flap (flap group) consisted of making the ILM flap and covering the MH with the flap or filling the flap into the MH. If necessary, an ophthalmic viscoelastic device was placed on the inverted ILM flap to prevent shifting. Sulfur hexafluoride (SF_6_) gas was used for tamponade in all cases. Cataract surgery was performed simultaneously when needed. After the operation, patients were directed to maintain the face-down position for about 1 week.

**Figure 1:**
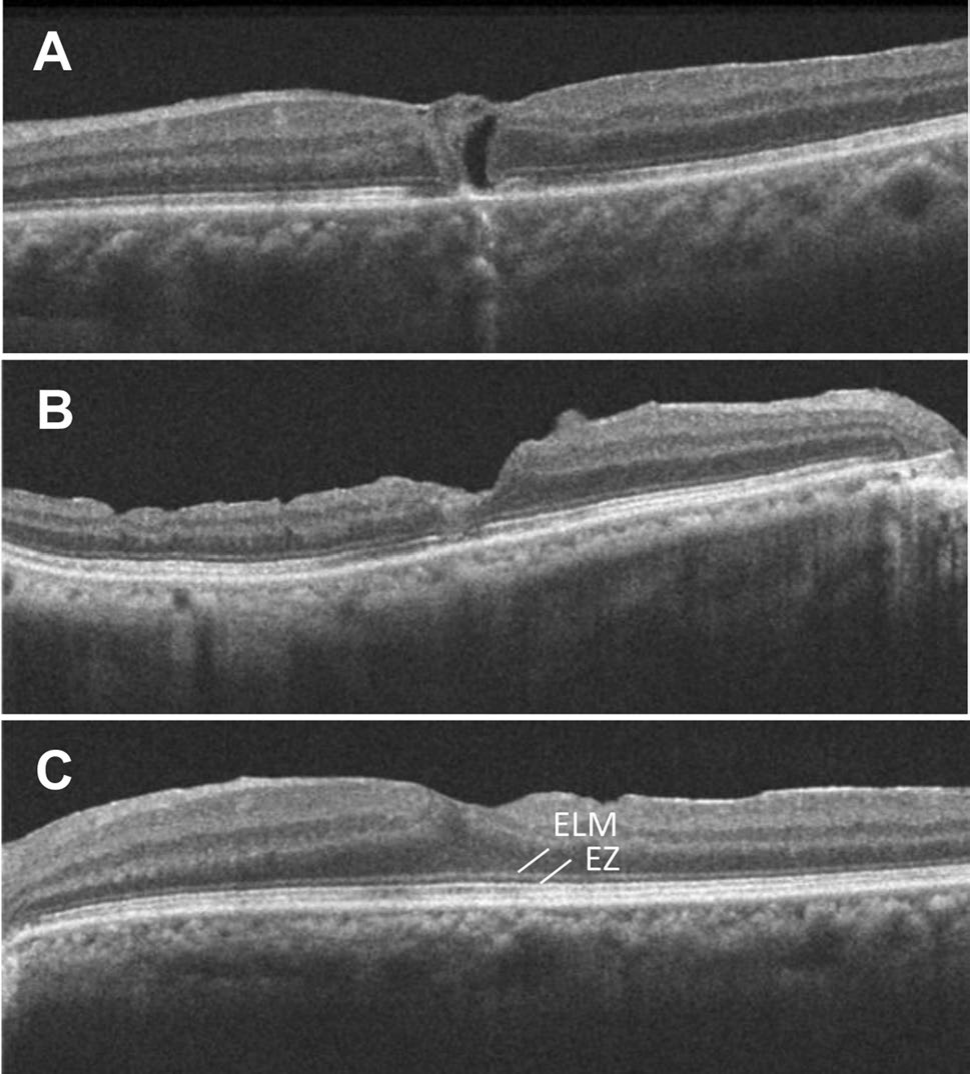
Recovery of the external limiting membrane (ELM) and ellipsoid zone (EZ) were assessed with a score using spectral-domain optical coherence tomography (SD-OCT). Score 0: defect, 1: indistinct, 2: continuous. (**A**) ELM: 0, EZ: 0 (**B**) ELM: 1, EZ:1 (**C**) ELM: 2, EZ: 2

The BCVA measurements and spectral domain OCT imaging (CirrusHD-OCT 5000, Carl Zeiss Meditec, Oberkochen, Germany) of horizontal and 5 raster scans covering a 6 mm × 6 mm area were performed in all patients at baseline and 1, 3, 6, and 12 months after the surgery. The BCVA was measured with a Landolt C chart and converted to logarithm of minimum angle of resolution (logMAR) equivalents for statistical analysis. At baseline, the minimum and the base diameters of the MH were measured manually using a built-in caliper. The minimum MH diameter was measured at the narrowest part of the MH, and the base diameter was measured at the base of the MH just above the pigment epithelium. After the surgery, recovery of the ELM and EZ were assessed within the central 2 mm area with a score of 0: defect, 1: indistinct, or 2: continuous (Fig. 1). Assessments were made by two doctors and discussed in case of mismatch.

### Statistical analysis

SPSS version 26 (IBM Japan, Tokyo, Japan) was used for statistical analysis.

To adjust for the selection bias for the procedure, we employed propensity score matching. Propensity score matching is a method that can be used to evaluate the effect of a treatment by accounting for the covariates that affect probability of receiving the treatment. The score is estimated with logistic regression analysis using variables presumed to be associated with both treatment and outcome. We can match each participant who underwent intervention to one or more participant who do not underwent the intervention. In the present study, we selected age, minimum MH diameter, MH base diameter, axial length, stages of MH, duration of symptoms, and preoperative BCVA as covariates.

One patient in the ILM peeling group was matched to one patient in the inverted ILM group based on the propensity score. Data are presented as frequencies for categorical variables and as means ± standard deviation or medians and interquartile range (IQR) (25th to 75th percentiles) for continuous variables.

The differences between two groups were assessed using Fisher’s exact test for categorical variables and Wilcoxon’s rank-sum test for continuous variables. The change in BCVA in each group was assessed using the Steel test. The primary outcome measure was postoperative BCVA, and the secondary outcome measures were MH closure and the recovery of ELM and EZ on OCT at the 12-month follow-up examination.

A value of P < 0.05 was considered significant.

## Results

Sixty-nine eyes of 68 patients were identified. After propensity score matching, 21 eyes of 21 patients in the peeling group were matched against 21 eyes of 21 patients in the flap group.

The baseline characteristics before and after propensity score matching of the flap group and the peeling group are shown in Table 1. There were no significant differences in age, preoperative BCVA, minimum MH diameter, base diameter, axial length, or duration of symptoms between the two groups before and after propensity score matching. The percentage of stage 3/4 MH eyes was higher in the flap group before propensity score matching, but this difference was not present after the matching, indicating that the two groups were successfully matched. Three eyes were already pseudo phakic in each group. Seventeen eyes in the flap group and 18 eyes in the peeling group underwent cataract surgery.

**Table 1:** Preoperative baseline characteristics before and after propensity score matching of the flap group and the peeling group.

	Pre matching				P	
	Flap n = 21		Peeling n = 48			
	(SD)	(IQR)	(SD)	(IQR)		
Age, median, years	66.2 (10.6)	68.0 (59.5–74.0)	66.1 (7.6)	66.0 (62.0–70.8)	0.88	
Pre-op BCVA, logMAR	0.70 (0.3)	0.70 (0.46–1.00)	0.66 (0.3)	0.70 (0.43–0.82)	0.35	
Snellen	20/100	20/100	20/91	20/100		
Minimum MH diameter, μm	278.6 (80.7)	288.0 (224.0–348.5)	252.3 (90.2)	269.0 (168.0–326.0)	0.27	
Base diameter, μm	686.0 (195.7)	704.0 (511.5–811.0)	667.0 (257.6)	644.5 (505.3–763.0)	0.39	
Axial length, mm	23.8 (1.2)	23.7 (22.6–25.02)	23.4 (1.3)	23.4 (22.5–24.2)	0.20	
Stage 3 or 4 (%)	19 (90.5)	19 (90.5)	32 (66.7)	32 (66.7)	0.04	
Duration of symptoms, m	2.5 (3.7)	2.0 (1–2.0)	2.1 (1.3)	2.0 (1.0–2.0)	0.44	

	Post matching				P	Standardized difference
	Flap n = 21		Peeling n = 21			
	(SD)	(IQR)	(SD)	(IQR)		
Age, median, y	66.2 (10.6)	68.0 (59.5–74.0)	66.6 (7.0)	67.0 (64.5–69.5)	0.94	0.045
Pre-op BCVA, logMAR	0.71 (0.3)	0.70 (0.46–1.0)	0.73 (0.3)	0.70 (0.52–0.91)	0.94	0.098
Snellen	20/102	20/100	20/107	20/100		
Minimum MH diameter, μm	278.6 (80.7)	288.0 (224.0–348.5)	276.0 (84.5)	288.0 (224.5–339.0)	0.89	0.031
Base diameter, μm	686.0 (195.7)	704.0 (511.5–811.0)	708.1 (276.6)	629.0 (570.0–764.5)	0.70	0.092
Axial length, mm	23.8 (1.2)	23.7 (22.6–25.0)	23.6 (1.4)	23.6 (22.7–24.5)	0.51	0.178
Stage 3 or 4 (%)	19 (90.5)	19 (90.5)	19 (90.5)	19 (90.5)	0.70	0
Duration of symptoms, m	2.4 (3.6)	2.0 (1.0–2.0)	2.3 (1.5)	2.0 (1.0–2.8)	0.25	0.061

Wilcoxon’s rank-sum test, Fisher’s exact test.

*BCVA* best-corrected visual acuity, *MH* macular hole, *logMAR* logarithm of minimum angle of resolution, *pre-op* pre-operative, *SD* standard deviation, *IQR* interquartile range (25th to 75th percentiles).

The MH was closed in all cases (21/21) in the peeling group. Meanwhile, anatomical closure was not achieved in 2 eyes (9.5%) in the flap group. In both cases, the inverted flap could not be confirmed on OCT taken 1 week after the surgery. We assumed that the flap was torn off or dislocated and failed to cover the MH. In one case, superior flap was employed in the first surgery and the remaining lower ILM was used to cover the MH. In the other case, there was no remaining ILM around the foveola. Thus, free ILM patch was taken from parafovea and was filled it into the MH. MH closure was achieved in both cases. There was no significant difference in the MH closure rate between the groups (P = 0.49).

**Figure 2:**
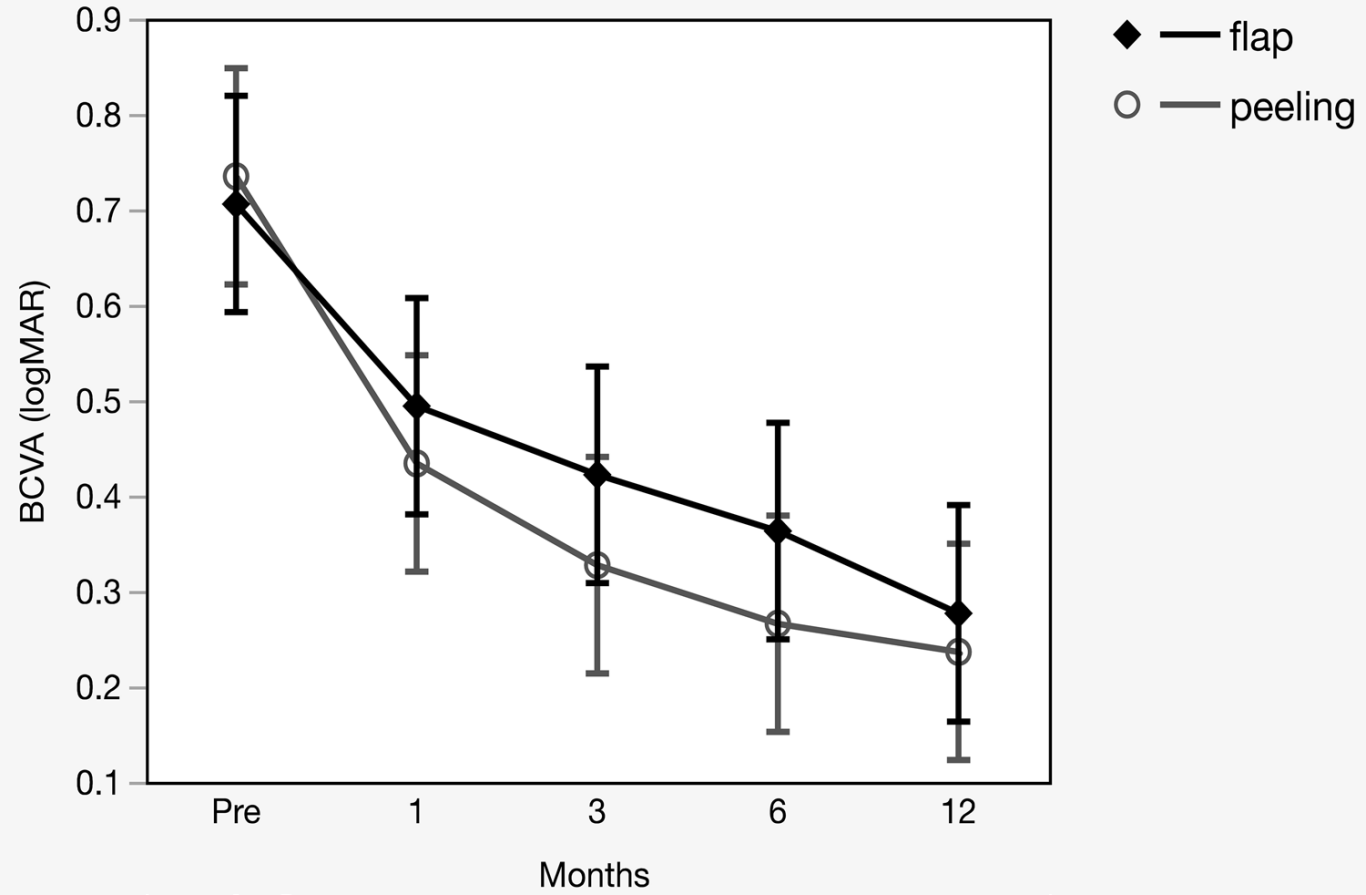
Change of pre-operative and postoperative best-corrected visual acuity (BCVA) in the flap group versus the peeling group. The change in BCVA in each group was assessed using the Steel test. Dot plots represent mean values, and whiskers represent 95% confidence intervals.

The visual outcome is shown in Table 2 and Fig. 2.

**Table 2:** Postoperative changes in BCVA and the MH closure rate of the flap group and the peeling group.

	Flap		Peeling		P
	n = 21		n = 21		
MH closure rate (%)	19 (90.5)		21 (100)		0.49

Post-op BCVA	logMAR (SD)	Snellen	logMAR (SD)	Snellen	
1 M	0.49 (0.33)	20/62	0.43 (0.21)	20/54	0.81
3 M	0.42 (0.30)	20/53	0.32 (0.17)	20/42	0.29
6 M	0.36 (0.26)	20/46	0.27 (0.18)	20/37	0.27
12 M	0.28 (0.24)	20/38	0.24 (0.24)	20/35	0.48

Wilcoxon’s rank-sum test, Fisher’s exact test.

*BCVA* best-corrected visual acuity, *MH* macular hole, *logMAR* logarithm of minimum angle of resolution, *SD* standard deviation, *post-op* postoperative.

In the flap group, a significant improvement was noted at 3 months after the surgery and thereafter. Meanwhile, the peeling group showed significant improvement of vision as early as one month after surgery. Nonetheless, there was no significant difference between the two groups in the postoperative BCVA at each time point (P = 0.81, P = 0.29, P = 0.27, and P = 0.48, respectively).

**Figure 3:**
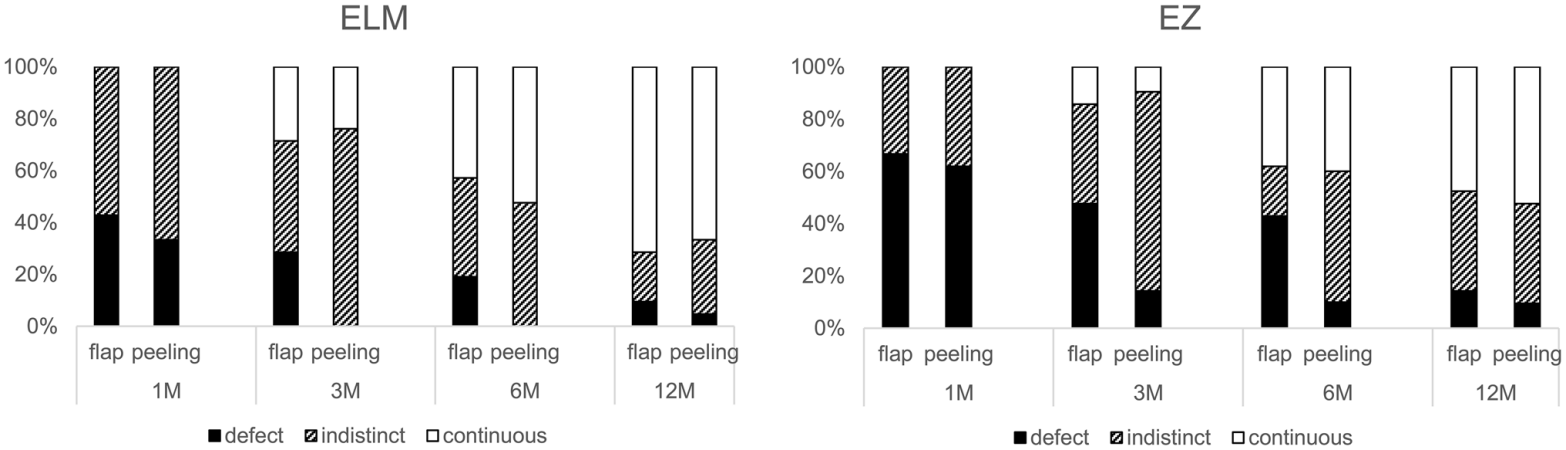
Recovery of the external limiting membrane (ELM) and ellipsoid zone (EZ) in the flap group and the peeling group at 1, 3, 6, and 12 months after surgery.

Recoveries of the ELM and EZ were evaluated using OCT images. Though both groups showed gradual recovery, the peeling group showed better anatomical status at 3 and 6 months after surgery (Fig. 3). The score for the ELM was higher in the peeling group at 3 months after surgery (P = 0.02), and the score for the EZ was higher in the peeling group at 3 and 6 months after surgery (P = 0.03, P = 0.04, respectively).

**Figure 4:**
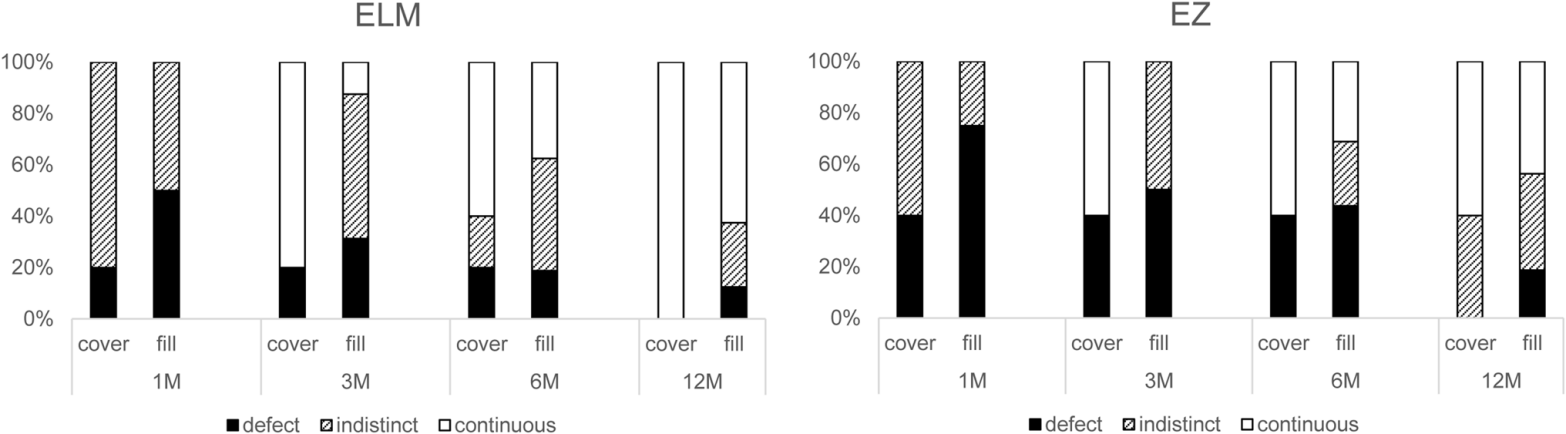
Recovery of the external limiting membrane (ELM) and ellipsoid zone EZ) in the cover group and the fill group at 1, 3, 6, and 12 months after surgery.

To investigate the reason for the delayed ELM and EZ recovery in the flap group, a subgroup analysis was performed by the invert method (cover: 5 eyes or fill: 16 eyes). There was no significant difference between the cover group and the fill group in the MH closure rate (80.0% vs 93.8%, P = 0.43). Meanwhile, improvement of BCVA was better in the cover group than in the fill group at 3 and 6 months after surgery (− 0.53 vs − 0.21, P = 0.047 and − 0.60 vs − 0.26, P = 0.047, respectively); this difference was not observed at 12 months after surgery (P = 0.17) (Table 3). The morphological changes of the ELM and EZ in the subgroups are shown in Fig. 4. The cover group had significantly higher scores for ELM and EZ recovery than the fill group at 3 months after the surgery (P = 0.01 and 0.004, respectively).

**Table 3:** Postoperative changes in BCVA and the MH closure rate of the cover group and the fill group.

	Cover		Fill		P
	n = 5		n = 16		
MH closure rate (%)	4 (80.0)		15 (93.8)		0.43

BCVA change	logMAR (SD)	Letters	logMAR (SD)	Letters	
1 M-pre	− 0.40 (0.3)	20	− 0.15 (0.3)	8	0.07
3 M-pre	− 0.53 (0.3)	27	− 0.21 (0.3)	11	0.047
6 M-pre	− 0.60 (0.3)	30	− 0.26 (0.3)	13	0.047
12 M-pre	− 0.65 (0.3)	33	− 0.36 (0.3)	18	0.17

Wilcoxon’s rank-sum test, Fisher’s exact test.

*BCVA* best-corrected visual acuity, *MH* macular hole, *logMAR* logarithm of minimum angle of resolution, *pre* pre-operative, *SD* standard deviation.

## Discussion

In the present study, conventional ILM peeling and inverted ILM flap technique were compared in eyes with small-medium size MHs (< 400 µm) using propensity score matching. The results showed that ILM flap technique, particularly ILM filling into the MH, delays functional and morphological recovery, though no difference was observed at 12 months after the surgery.

In the present study, propensity score matching was used to adjust baseline factors between the peeling and flap groups. Whereas both groups showed similar characteristics even before propensity score matching, there was a slight difference in the percentage of stage 3/4 cases. Propensity score matching successfully adjusted the difference.

The MH closure rate was not different between the flap and peeling groups, probably because the closure rate is already high in conventional peeling. Visual outcome was not different between the two groups as well. However, improvement in BCVA was slower in the flap group; visual improvement at one month after surgery was significant in the peeling group, but not in the flap group. Evaluation of OCT images also showed a tendency for slower recovery of outer retinal integrity in the flap group. There were significant differences in ELM and EZ recovery at three and six months after the surgery.

Many variations of the inverted ILM flap technique have been reported to date^21–23^ since Michalewska et al.^9^ reported it using temporal ILM flap. Several reports dealing with large MHs also showed that the recovery of the outer retinal layer is delayed with inverted ILM flap compared to conventional ILM peeling^24, 25^. These results indicate that the inverted ILM flap technique is associated with delayed functional and morphological recovery. Further, some reports^26–29^ found that the cover technique outperformed the fill-in technique in functional outcomes. The present study included patients who underwent surgery between 2014 and 2017. At that time, these variations were not well established, and the present study includes many cases who underwent fill-in technique. That may partly explain the slow recovery in ILM flap group.

There can be some explanations for the delayed recovery after ILM flap technique, particularly fill-in technique. Shiode et al.^30^ reported that the ILM functions as a scaffold for the proliferation and migration of Müller cells, and it may promote Müller cell activation. Migrating Müller cells secrete neurotrophic factors that may promote glial hypertrophy. It is suggested that this glial hypertrophy contributes to MH closure. The inverted ILM flap technique, particularly when the ILM flap is filled into the MH, may promote glial hypertrophy in the space that should be filled with neural retina and may inhibit extension of the ELM and EZ. Delayed morphological recovery after ILM filling was also suggested in a previous study^31^. Although the technique is certainly useful for refractory cases such as large MH and MH retinal detachment, the promoted glial hypertrophy may be superfluous for small/medium size.

This study has some strengths and limitations. Propensity score matching was used, and the outcomes were compared in baseline-matched cohorts. Thus, the observed differences can be attributed to the difference in surgical procedures. The selection of the surgical technique was at the surgeon’s discretion and many cases underwent fill-in technique, which is a limitation of this comparative study. Other limitations include the retrospective design and the small sample size. The results need to be confirmed in other cohorts.

In conclusion, there was no significant difference between the flap group and the peeling group in the postoperative BCVA at each time point. The ILM flap technique, particularly ILM fill technique, for small-medium size MHs has no significant beneficial effect on the MH closure rate and may delay the recovery of the outer retinal layer compared to the conventional ILM peeling technique. Surgeons should note that the ILM flap technique is not a panacea for all MHs. The appropriate technique should be applied for each case
